# Alcohol and tobacco use and risk of multiple myeloma: A case‐control study

**DOI:** 10.1002/jha2.337

**Published:** 2021-11-10

**Authors:** Simon Cheah, Julie K. Bassett, Fiona J. Bruinsma, Wendy Cozen, John L. Hopper, Harindra Jayasekara, Douglas Joshua, Robert J. MacInnis, H. Miles Prince, Claire M. Vajdic, Marina T. van Leeuwen, Nicole Wong Doo, Simon J. Harrison, Dallas R. English, Graham G. Giles, Roger L. Milne

**Affiliations:** ^1^ Cancer Epidemiology Division Cancer Council Victoria Melbourne Australia; ^2^ Centre for Epidemiology and Biostatistics Melbourne School of Population and Global Health University of Melbourne Melbourne Australia; ^3^ Department of Preventive Medicine University of Southern California Los Angeles California USA; ^4^ Royal Prince Alfred Hospital Sydney Medical School University of Sydney Sydney Australia; ^5^ Sir Peter MacCallum Department of Oncology University of Melbourne Melbourne Australia; ^6^ Epworth Healthcare Melbourne Australia; ^7^ Centre for Big Data Research in Health The University of New South Wales Sydney Australia; ^8^ Concord Clinical School University of Sydney Sydney Australia; ^9^ Clinical Haematology Peter MacCallum Cancer Centre and Royal Melbourne Hospital Parkville Australia; ^10^ School of Clinical Sciences at Monash Health Precision Medicine Monash University Clayton Melbourne Australia

**Keywords:** alcohol, epidemiology, family case–control study, multiple myeloma, smoking

## Abstract

Multiple myeloma (MM) is the second most common hematological cancer and causes significant mortality and morbidity. Knowledge regarding modifiable risk factors for MM remains limited. This analysis of an Australian population‐based case–control family study investigates whether smoking or alcohol consumption is associated with risk of MM and related diseases. Incident cases (*n* = 789) of MM were recruited via cancer registries in Victoria and New South Wales. Controls (*n* = 1,113) were either family members of cases (*n* = 696) or controls recruited for a similarly designed study of renal cancers (*n* = 417). Adjusted odds ratios (OR) and 95% confidence intervals (CI) were estimated using unconditional multivariable logistic regression. Heavy intake (>20 g ethanol/day) of alcohol had a lower risk of MM compared with nondrinkers (OR = 0.68, 95% CI: 0.50–0.93), and there was an inverse dose–response relationship for average daily alcohol intake (OR per 10 g ethanol per day = 0.92, 95% CI: 0.86–0.99); there was no evidence of an interaction with sex. There was no evidence of an association with MM risk for smoking‐related exposures (*p* > 0.18). The associations between smoking and alcohol with MM are similar to those with non‐Hodgkin lymphoma. Further research into potential underlying mechanisms is warranted.

## 1 INTRODUCTION

Multiple myeloma (MM) is a plasma cell neoplasm arising from the malignant transformation of mature postgerminal center B cells [1]. MM is typically preceded by the asymptomatic precursor condition monoclonal gammopathy of undetermined significance (MGUS), which progresses to multiple myeloma at an average rate of 1% per year [2]. Despite improvements in survival, multiple myeloma remains essentially incurable [3]. It accounts for 10%–15% of all hematological cancers [4 ^–^ 6], and represents a disproportionately large fraction of all cancer mortality: 2.2%, compared with all cancer incidence: 1.6%, in Australia [7].

Few risk factors for multiple myeloma have been firmly established, and most known risk factors are nonmodifiable; advanced age [8], male sex [4], black African ancestry, and positive family history [9] have all been linked to increased MM risk. Apart from certain chemical and occupational exposures [10 ^–^ 12], body mass index (BMI) is the only well‐established modifiable risk factor for MM [13].

Although lifestyle factors such as tobacco smoking and alcohol use are responsible for a large proportion of cancers [14], research on common modifiable risk factors for MM has been limited by the inability of smaller observational studies to detect effects of public health significance [4]. Large observational studies and meta‐analyses have indicated that while tobacco smoking is unlikely to be associated with MM risk [15 ^–^ 17], alcohol consumption may be inversely associated [15, 18 ^–^ 22], and this inverse association is possibly stronger for women, and wine drinkers [20, 23]. Observational studies investigating non‐Hodgkin lymphoma risk have identified a similar inverse association with alcohol [24, 25].

The aim of this study is to investigate whether tobacco and alcohol consumption are associated with the risk of MM, and whether the association between alcohol and MM is modified by sex.

## 2 SUBJECTS AND METHODS

### 2.1 Study population and recruitment

To investigate the effect of these common modifiable risk factors on MM risk, we conducted an analysis using the Epidemiology of Multiple Myeloma in Australia (EMMA) study. EMMA is a population‐based, family‐based, case–control study designed to examine the effect of modifiable exposures on MM risk (and identify associated genetic factors).

The EMMA study recruited newly diagnosed cases of MM or MGUS aged 20–74 years residing in Victoria or New South Wales (NSW). The eligibility criteria are provided in Table 1. Cases were primarily recruited via the corresponding state cancer registries, with additional recruitment via clinicians from hospitals, clinics and patient support groups. Family members of cases were recruited as controls. The Cancer Council Victoria Human Research Ethics Committee and NSW Population and Health Services Research Ethics Committee approved this study. All participants gave written informed consent.

**TABLE 1:** Summary of epidemiology of multiple myeloma in Australia (EMMA) study eligibility criteria for case and control recruitment

Case eligibility criteria Age at diagnosis between 20 and 74 years Histologically confirmed multiple myeloma (ICD‐O‐3: M9732/3) recruited within 12 months of diagnosis, OR clinical diagnosis of monoclonal gammopathy of unknown significance or other myeloma‐related condition such as smouldering myeloma May have had previous primary invasive cancer Resident of Victoria, NSW or Queensland, Australia Able to complete questionnaires in English Able to give informed consent
Control eligibility criteria Relative or family member of a case No history of haematological malignancy Able to complete questionnaires in English Able to give informed consent Preference to Australian residents

Victorian and NSW state‐based cancer registries identified all new diagnoses of MM and plasmacytoma (ICD‐O‐3: M9732/3) during the study period; histologically confirmed incident cases of MM were recruited if diagnosed between 1 January 2010 in Victoria, or 1 January 2013 in NSW, and 31 December 2016. All cancer diagnoses in Australia must, by law, be notified to a state‐based cancer registry; registration was, therefore, considered to be virtually complete [26]. MGUS (ICD10 D47.2) must also be reported by statute to the Victorian Cancer Registry; MGUS cases in NSW were recruited through clinicians.

Once identified as eligible, treating physicians were informed of the intention to invite their patients to participate. An invitation, information sheet and study consent form were sent to the individual, unless the treating practitioner declined participation. Thereafter, the registry released contact details of consenting individuals to the research team.

Individuals diagnosed with MM and related conditions such as MGUS between mid‐2012 and 31 December 2016 were eligible for clinic‐based recruitment. Clinicians informed potentially eligible individuals of the study and provided them with a study brochure and expression of interest (EOI) form. After receiving an EOI, researchers reviewed their eligibility before inviting them to participate.

During recruitment, cases were asked for consent to invite family members to participate as controls (Table 1). The EMMA study aimed to recruit as controls at least one family member selected from living relatives unaffected by hematological malignancy; preference was for the same‐sex sibling closest in age to the case, followed by any sibling of the same sex, and if available, the case's spouse or partner was also recruited. Multiple sibling controls were recruited from some families to help balance numbers for those cases without siblings.

We also included additional controls from the Consortium for the Investigation of Renal Malignancies (CONFIRM) study, a case–control family study of renal cancer with a similar design and questionnaire (see Supporting Information 1).

### 2.2 Data collection

Consenting cases and controls completed self‐administered questionnaires on lifestyle, health and medical history, family history, residential and occupational history, and diet [27].

### 2.3 Statistical analysis

Alcohol‐related exposures investigated include average daily ethanol intake (continuous and categorical) and beverage type. Average ethanol intake in grams per day was calculated based on reported frequency, quantity and type of alcoholic beverages consumed in the year starting 2 years prior to interview. For each type of alcoholic beverage, we converted frequencies to daily equivalents and estimated the volume consumed in grams per day. The ethanol intake in grams per day was then estimated using the average ethanol content per 100 g of each type of alcoholic beverage from the Australian Food Composition Database [28]. Grams of ethanol for each beverage type were summed to give daily average ethanol intake overall, and for each of wine, beer, and spirits separately. Participants were subsequently categorized as nondrinkers (0 g/day), moderate drinkers (1–20 g/day), or heavy drinkers (>20 g/day) based on the National Health and Medical Research Council guidelines current at the time of recruitment and data collection (one Australian standard drink contains 10 g of pure ethanol) [29].

For tobacco, we investigated smoking status, pack‐years (including an ever‐smoking indicator), smoking duration, and intensity[30]. Participants were categorized according to smoking status (never vs. ever – at least seven cigarettes/week for a year, and forever smokers, current vs. former smokers – ceased smoking at least 2 years prior), duration of use (total years, mean‐centered), smoking intensity (average cigarettes per day, mean‐centered), mean‐centered pack‐years ((duration × intensity)/20), age at initiation (years), and time since cessation (years).

In the primary analysis cases of MM and related diseases including MGUS were combined, as were controls from EMMA and CONFIRM studies.

### 2.4 Covariates

Covariates included in the primary analyses included sex, age (continuous), state (Victoria or New South Wales), and country of birth (Australia/New Zealand, Europe/UK, or other). Analyses examining alcohol exposures were additionally adjusted for smoking status, and smoking analyses were adjusted for alcohol intake (continuous). Covariates for inclusion in our models were selected based on the literature and following causal diagram analysis (Supporting Information 3).

The risk of MM associated with various exposures was estimated using unconditional multivariable logistic regression to estimate odds ratios (OR) and 95% confidence intervals (CI). All statistical tests were two‐sided and *p*‐values < 0.05 were considered statistically significant. Robust standard errors were used to account for clustering within sibships. To investigate the effect of beverage type while holding total alcohol intake constant, we estimated beverage‐specific substitution effects: for example, the effect of substituting one additional standard drink (10 g ethanol) per day of wine for one fewer standard drink of other alcohol on MM risk, by subtracting the regression coefficient for the estimated effect of nonwine alcoholic beverages, from the regression coefficient for wine [31]. We assessed potential two‐way interactions between sex and alcohol intake using the Wald‐test for the interaction term in models for sex and alcohol consumption. Participants missing data for key exposures or covariates were excluded from the analysis (Figure 2).

Sensitivity analyses were performed adjusting for BMI (continuous), since there is some evidence for bidirectionality in the associations between BMI and both alcohol and smoking [32 ^–^ 35]. We also performed sensitivity analyses restricted to EMMA study participants to assess potential bias that may be introduced by control selection, matching or strong familial correlation for risk factors: (1) unmatched: including all cases and all controls; (2) unmatched: all cases but including only spouse controls; (3) matched: including cases with their one or more matched sibling controls; and (4) matched: 1:1 including cases with their matched spouse control. For matched analyses, conditional logistic regression was used to estimate ORs and 95% confidence intervals. All analyses were conducted using Stata/MP 16.1 for Windows (StataCorp LLC, USA).

## 3 RESULTS

**FIGURE 1:**
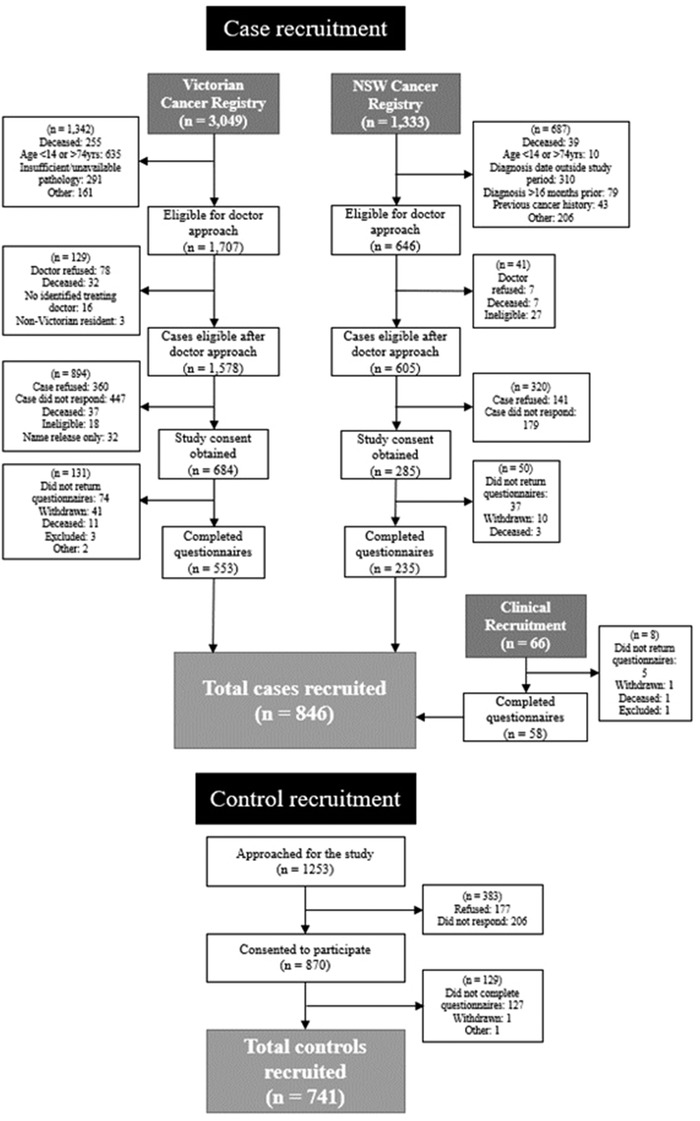
Flowchart: Recruitment of epidemiology of multiple myeloma in Australia (EMMA) cases and controls

**FIGURE 2:**
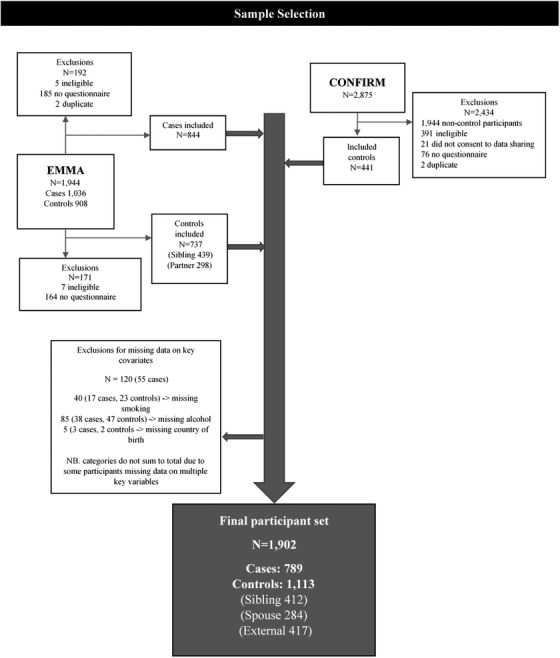
Sample selection flowchart

Consent was obtained from 969 (44%) of 2183 eligible registry‐sourced cases, and 66 clinic‐based cases (Figure 1). Of all consented cases, 846 (82%) returned questionnaire data, with 789 (76%) included in the analysis after exclusions based on missing data (Figure 2); 67 MGUS cases were included in the final analysis. For cases, the median time from diagnosis to questionnaire completion was 9.6 months.

Of the 1253 potential family controls approached for inclusion in EMMA, 870 (69%) consented to participate and 741 (59%) returned completed questionnaires. Of those participants returning questionnaires, seven control participants were ineligible due to a history of previous hematological cancer, and 41 were excluded due to missing data on key covariates, leaving 696 controls available for analysis. Of the 834 controls in CONFIRM who returned questionnaires and consented to data‐sharing, 391 were ineligible either due to residing outside of NSW or Victoria (*n* = 383) or a personal history of hematological cancer (*n* = 8), and another 26 were excluded based on missing data, leaving 417 (50%) controls available for analysis.

Table 2 summarizes the characteristics of cases and controls. Men comprised 57% cases and 40% controls. The average age at diagnosis of MM was 60.9 years, while the mean age at questionnaire completion was 62.9 years for cases and 61.4 years for controls. Controls were more likely than cases to be born in Australia or New Zealand. Victorian participants accounted for most of the cases and controls. Cases were less likely to be never smokers but more likely to be nondrinkers compared with controls.

**TABLE 2:** Descriptive characteristics for cases and controls overall and by study

Characteristic	Cases		Controls		EMMA controls		CONFIRM controls	
N (%)	789	(41.5)	1113	(58.5)	696		417	
Age at Qx completion, mean (SD)	62.9	(8.6)	61.4	(9.0)	62.1	(8.4)	60.3	(9.8)
Age at diagnosis, mean (SD)	60.9	(9.6)		(.)		(.)		(.)
BMI (kg/m^2^), mean (SD)	27.1	(4.7)	27.4	(5.3)	27.1	(5.2)	27.8	(5.4)
State, N (%)								
Victoria	549	(69.6)	872	(78.3)	455	(65.4)	417	(100.0)
New South Wales	240	(30.4)	241	(21.7)	241	(34.6)		(.)
Sex, N (%)								
Female	340	(43.1)	663	(59.6)	398	(57.2)	265	(63.5)
Male	449	(56.9)	450	(40.4)	298	(42.8)	152	(36.5)
Alcohol consumption, N (%)								
Non‐drinker	134	(17.0)	161	(14.5)	99	(14.2)	62	(14.9)
Moderate (≤20 g)	488	(61.9)	703	(63.2)	429	(61.6)	274	(65.7)
Heavy (>20 g)	167	(21.2)	249	(22.4)	168	(24.1)	81	(19.4)
Smoking status, N (%)								
Never	436	(55.3)	651	(58.5)	401	(57.6)	250	(60.0)
Former	288	(36.5)	376	(33.8)	242	(34.8)	134	(32.1)
Current	65	(8.2)	86	(7.7)	53	(7.6)	33	(7.9)
Family history (first deg), N (%)								
No	728	(92.3)	254	(22.8)	254	(36.5)		(.)
Yes	20	(2.5)	321	(28.8)	321	(46.1)		(.)
Unknown	41	(5.2)	538	(48.3)	121	(17.4)	417	(100.0)
Country of birth, N (%)								
Australia or New Zealand	582	(73.8)	931	(83.6)	576	(82.8)	355	(85.1)
Europe (including UK)	139	(17.6)	133	(11.9)	83	(11.9)	50	(12.0)
Other	68	(8.6)	49	(4.4)	37	(5.3)	12	(2.9)

### 3.1 Alcohol

**FIGURE 3:**
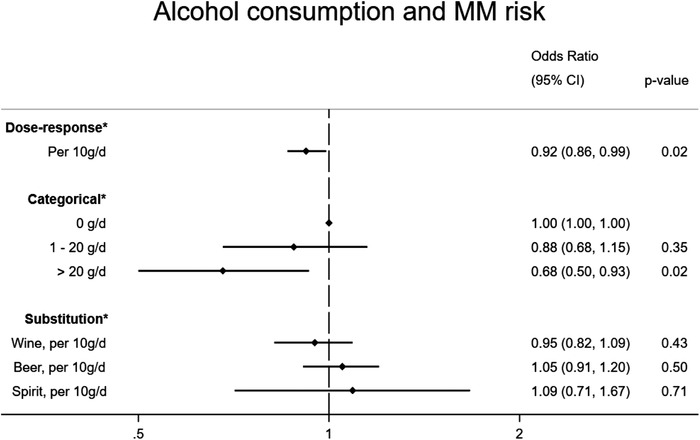
Alcohol consumption and multiple myeloma risk

**FIGURE 4:**
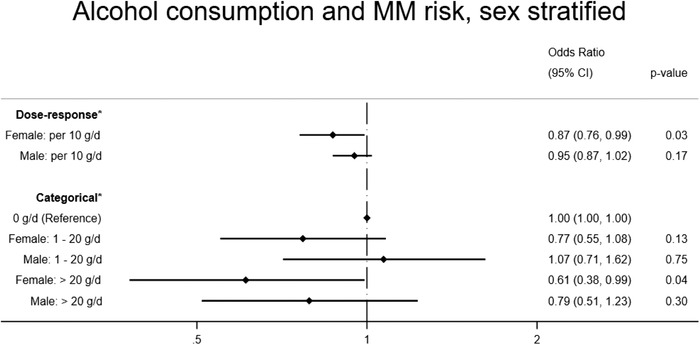
Alcohol consumption and multiple myeloma risk, stratified by sex

Table 3, and Figures 3 and 4, present results for alcohol‐related exposures. Compared with no consumption, consuming more than 20 g of alcohol per day was associated with an estimated 32% reduced risk of MM (OR = 0.68, 95% CI: 0.50–0.93, *p* = 0.02), but no association was observed for moderate drinkers (OR = 0.88, 95% CI: 0.68–1.15, *p* = 0.35). For each 10 g ethanol/day increase in average alcohol intake, the risk of MM decreased by 8% (OR = 0.92, 95% CI: 0.86–0.99, *p* = 0.02).

**TABLE 3:** Alcohol consumption and risk of multiple myeloma

	Adjusted* OR	(95% CI)	P‐value	Cases	Controls
Total				789	1113
Drinking category					
Nondrinker (0 g/day)	1.00			134	161
Moderate drinker (1–20 g/day)	0.88	(0.68, 1.15)	0.35	488	703
Heavy drinker (>20 g/day)	0.68	(0.50, 0.93)	0.02	167	249
Per standard drink (10 g ethanol) per day	0.92	(0.86, 0.99)	0.02	789	1113
Beverage substitution effect				789	1113
Wine	0.95	(0.82, 1.09)	0.43		
Beer	1.05	(0.91, 1.20)	0.50		
Spirit	1.09	(0.71, 1.67)	0.71		
Alcohol consumption by sex					
Drinking category (females)					
Nondrinker (0 g/day)	1.00			74	106
Moderate drinker (1–20 g/day)	0.77	(0.55, 1.08)	0.13	224	460
Heavy drinker (>20 g/day)	0.61	(0.38, 0.99)	0.04	42	97
Drinking category (males)					
Nondrinker (0 g/day)	1.00			60	55
Moderate drinker (1–20 g/day)	1.07	(0.71, 1.62)	0.75	264	243
Heavy drinker (>20 g/day)	0.79	(0.51, 1.23)	0.30	125	152
Wald test for interaction			0.49		
Alcohol consumption (standard drinks per day)					
OR for increase in 1 standard drink per day for women	0.87	(0.76, 0.99)	0.03	340	663
OR for increase of 1 standard drink per day for men	0.95	(0.87, 1.02)	0.17	449	450
Wald test for interaction			0.27		

* Drinking category defined by average daily ethanol intake in grams (g/d), non‐drinker: 0 g/d, moderate drinker: 1–20 g/d, heavy drinker > 20 g/d. Dose–response per 10 g/d daily average ethanol intake. Beverage substitution effect: predicted change in odds associated with substituting one more standard drink per day of this type while holding total alcohol intake constant. Odds ratios in continuous and categorical analyses adjusted for age, sex, state, country of birth, and smoking status (never/former/current).

Based on the beverage substitution analysis, we found no substantial change in risk of MM associated with increasing one standard drink per day of wine (OR = 0.95, 95% CI: 0.82–1.09, *p* = 0.43), beer (OR = 1.05, 95% CI: 0.91–1.20, *p* = 0.39) or spirits (OR = 1.09, 95%CI: 0.71–1.67, *p* = 0.58), keeping total ethanol intake constant [31]. We observed no evidence of an interaction between sex and alcohol consumption, modeled continuously (*p* = 0.27) or categorically (*p* = 0.49), despite observing lower ORs for women (OR = 0.87, 95% CI: 0.76–0.99) compared with men (OR = 0.95, 95% CI: 0.87–1.02) per standard drink per day increase in alcohol intake.

### 3.2 Tobacco

**FIGURE 5:**
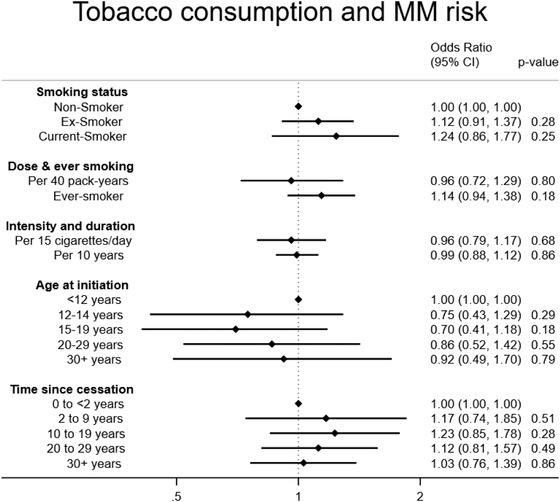
Tobacco consumption and multiple myeloma risk

Table 4 and Figure 5 shows results for tobacco‐related exposures. Compared with nonsmokers, we found no clear evidence of association with risk of MM for either former (OR: 1.12, 95% CI: 0.91–1.37) or current (OR = 1.24, 95% CI: 0.86–1.77) smoking. Nor did we find evidence of association for smoking pack‐years (OR = 0.96, 95% CI 0.72–1.29, per 40 pack‐years), ever‐smoking (OR = 1.14, 95% CI: 0.94–1.38), smoking duration (OR = 0.99 per 10 years smoking duration, 95% CI: 0.88–1.12) or smoking intensity (OR = 0.96 per 15 cigarettes per day, 95% CI: 0.79–1.17), time since smoking cessation, or years since smoking initiation.

**TABLE 4:** Tobacco smoking and risk of multiple myeloma

	Adjusted OR*	(95% CI)	*p*‐value	Cases	Controls
Smoking status					
Nonsmoker	1.00			436	651
Former smoker	1.12	(0.91, 1.37)	0.28	288	376
Current smoker	1.24	(0.86, 1.77)	0.25	65	86
Pack‐year history*^#^* (per 40 pack‐years)	0.96	(0.72, 1.29)	0.80	789	1113
Ever‐smoker	1.14	(0.94, 1.38)	0.18	789	1113
Smoking duration (per 10 year increment)	0.99	(0.88, 1.12)	0.86	789	1113
Smoking intensity (per 15 cigarettes/day)	0.96	(0.79, 1.17)	0.68	789	1113
Time since cessation 0 to < 2	1.00			501	737
2–10	1.17	(0.74, 1.85)	0.51	38	54
10–20	1.23	(0.85, 1.78)	0.28	60	79
20–30	1.12	(0.81, 1.57)	0.49	72	94
30+ years	1.03	(0.76, 1.39)	0.86	118	149
Age at initiation <12 years	1.00			556	804
12–14	0.75	(0.43, 1.29)	0.29	25	42
15–19	0.70	(0.41, 1.18)	0.18	32	56
20–29	0.86	(0.52, 1.42)	0.55	71	86
30+ years	0.92	(0.49, 1.70)	0.79	105	125

* Adjusted for age, sex, state, country of birth, and alcohol consumption (continuous).

^#^One pack‐year equivalent to smoking 20 cigarettes (i.e., one “pack”) per day for 1 year, 40 pack‐years would be equivalent to doing the same for 40 years (or, e.g., smoking two packs per day for 20 years). Pack‐years, duration and intensity were all mean‐centered. Analyses included all eligible participants.

### 3.3 Sensitivity analyses

Sensitivity analyses for alcohol and smoking exposures were generally consistent with the primary findings (see Supporting Information 2). This was true for those sensitivity analyses using conditional logistic regression, in matched sets of cases either with spouses, or with siblings, as well as in unconditional regression analyses excluding siblings (EMMA participants only) or restricted to non‐EMMA controls. There were no substantial differences in results from analyses adjusting for BMI.

## 4 DISCUSSION

In this large case–control family study, we observed an inverse association of alcohol consumption with MM risk, both for heavy drinkers relative to nondrinkers and with increasing alcohol consumption. However, we found no conclusive evidence of differences in this association by sex. Nor did we find any substantial association between tobacco usage and myeloma risk.

Despite accumulating epidemiological evidence for this association, the biological mechanism by which alcohol consumption might reduce MM risk is not yet understood. It has been suggested that low alcohol intake can improve insulin sensitivity, and thus might indirectly influence the risk of MM via diabetes‐ or obesity‐linked mechanisms [36]. Mammalian target of rapamycin (mTOR) signaling, a target relevant for MM tumorigenesis[37], was found to be inhibited in human lymphoma xenograft models by chronic low dose ethanol [38]. Others have suggested that resveratrol, found in grape skin and red wine, could reduce MM risk, despite its low oral bioavailability [39 ^–^ 41]. In vitro, resveratrol has demonstrated inhibition of STAT3 and NF‐κВ, suppression of MM cell proliferation and potentiation of the apoptotic effect of bortezomib [42, 43]. Other polyphenols found in wine, beer, and dark spirits have also demonstrated anti‐tumorigenic properties via NF‐κВ and other pathways in MM cells, and differences in phenolic content and concentration could potentially contribute to the previously reported differences in association with myeloma risk by alcoholic beverage type [44, 45]; however, alcoholic beverage type substitution analyses did not support these hypotheses. Another potential mechanism is via the effect of alcohol on inflammatory markers; moderate to high alcohol consumption (15–30 g/day) is inversely associated with circulating interleukin‐6 (IL‐6), a cytokine which can stimulate the growth of myeloma cells and has been associated with poor prognosis, and circulating C‐reactive protein (CRP) a surrogate for IL‐6 [46 ^–^ 48].

While other studies have suggested there may be a stronger inverse association for women compared with men for alcohol consumption and MM risk [18, 20, 21], this study did not demonstrate statistical interaction between alcohol and sex, despite finding larger inverse effect sizes for alcohol consumption and MM risk for women. Substantially larger samples may be necessary to convincingly infer or exclude interaction.

In the primary analysis, we identified no substantial associations for smoking status, pack‐year history, time since cessation, smoking duration, age at smoking initiation, or smoking intensity with risk of MM. This general lack of association is consistent with most epidemiological literature investigating smoking and MM [4, 16, 17, 23].

A strength of this study is its family‐based design, with stronger motivation for control participation, potential reductions in volunteer and recall bias [49] and the improved cost‐effectiveness of an integrated recruitment process [50]. With volunteer controls sourced from the general population, it is becoming increasingly difficult to achieve satisfactory response rates [51]. Other strengths of this study include a large incident case population, adjustment for known confounders in the analysis, and the use of sibling controls which could reduce confounding by unmeasured early life or genetic factors [52].

One limitation of this study was our inability to completely differentiate lifetime alcohol abstainers from those who may have been prompted to more recent intake reduction. Although the study examined a historical alcohol‐exposure window 2 years prior to questionnaire, alcohol‐intake reduction is often associated with ill health and advancing age, and as such we cannot entirely exclude reverse causation or residual confounding bias in our results [53]. Another limitation was the inability to directly examine ethnicity. Individuals of African ancestry have been found to have an elevated risk of MM. However, this could not be adequately examined due to an insufficient number of African‐background participants. We found the overseas country of birth to be associated with increased MM risk, which indicates that some early life exposures or genetic factors may potentially contribute to MM risk. Due to age restriction, results may not apply to individuals aged 75 or older.

The family‐based design also has some inherent limitations; exposures tend to be correlated within families, which means that family‐based studies may have less power to detect certain associations than similar studies with unrelated controls.[54] We might expect this to be pronounced especially for sibling‐controls with shared genetic and early‐life exposures, and perhaps with shared later‐life socio‐environmental exposures for spouse‐controls, disregarding potential assortative mating [49, 50]. Given that tobacco and alcohol consumption are complex traits, for which there is evidence for both genetic and environmental influences, this could have affected precision [55 ^–^ 58]. Yet the findings of this family‐based study were similar to those from previous studies using population controls, even when unrelated controls were excluded from the analysis. This suggests that the simultaneous use of multiple types of familial controls might mitigate against statistical inefficiency for certain exposures.

## 5 CONCLUSION

To our knowledge, EMMA is the first multi‐center case–control study to investigate the epidemiology of MM in Australia. This study extends the evidence base for alcohol, tobacco, and MM risk by examining a novel Australian study population, and the use of family controls complements previous findings from other observational study designs [16, 20].

Although this study finds an inverse association between alcohol consumption and MM risk, we do not recommend alcohol consumption as a measure for MM prevention as other studies have found that any level of alcohol consumption increases overall cancer risk and all‐cause mortality [59].

Further research investigating the potential causality and mechanisms underlying the observed MM‐alcohol association is recommended. While a randomized controlled trial would be inappropriate due to ethical considerations, other designs, such as Mendelian randomization, could provide additional insight into causality.

## CONFLICT OF INTEREST

The authors declare no conflict of interest.

## Supporting information

SUPPORTING INFORMATION
Additional information may be found online in the Supporting Information section at the end of the article.

## Data Availability

The data that support the findings of this study are not publicly available due to privacy or ethical restrictions.
