# Supplementary material for: Alcohol and tobacco use and risk of multiple myeloma: A case‐control study
Source: EJHaem. 2021 Nov 10;3(1):109–20. doi: 10.1002/jha2.337 (PMC9175849; doi:10.1002/jha2.337)
Supplement: Supplementary file 1 — SUPPORTING INFORMATION Additional information may be found online in the Supporting Information section at the end of the article. [file JHA2-3-109-s001.docx]

# SUPPORTING INFORMATION

## Supporting information 1: Comparison of EMMA and CONFIRM study characteristics

**Table S1.1. Comparison of EMMA and CONFIRM study characteristics**

| **Study** | **EMMA** | **CONFIRM** |
| --- | --- | --- |
| *Source* | EMMA Protocol v10 (Feb 2015) | CONFIRM Protocol v4.0 (July 2014) |
| *Design* | Family case-control | Family case-control |
| *Recruitment State/Territory* | VIC  NSW | VIC  QLD* |
| Time period (recruitment) | 2010-2016  *(VIC 2010-2016, NSW 2013-2016) | 2011-2014 |
| *Location* | Preference given to Australia resident over overseas resident | NA* |
| *Control recruitment/Relationship to case* | Sibling or partner of case | Family member of case |
| *Exclusions for previous cancer* | Previous haematological malignancy excluded | NA* |
| *Age/Sex* | preferably age-and sex-matched sibling to case, or partner if no appropriate siblings  NB. case must be Dx between 20-75 years | Case must be Dx between 18-75 years  M&F |
| *Language* | Able to complete questionnaires in English | Able to complete questionnaires in English |
| *Consent* | Able to give informed consent | Able to give informed consent |

*NB in the present analysis, CONFIRM controls were restricted to Victoria and those with a history of haematological malignancy were excluded.

## Supporting information 2: Sensitivity analyses

**Table S2.1. Alcohol consumption and risk of multiple myeloma, adjusted for BMI**

|  | **OR*** | **(95% CI)** | **P-value** | **Cases** | **Controls** |
| --- | --- | --- | --- | --- | --- |
| **Total** |  |  |  | 767 | 1077 |
| **Drinking category** |  |  |  |  |  |
| *Non-drinker (0g/day)* | 1.00 |  |  |  |  |
| *Moderate drinker (0-20g/day)* | 0.84 | (0.64, 1.11) | 0.22 |  |  |
| *Heavy drinker (>20g/day)* | 0.63 | (0.46, 0.87) | 0.01 |  |  |
|  |  |  |  |  |  |
| **Per standard drink (10g ethanol) per day** | 0.91 | (0.85, 0.98) | 0.01 |  |  |
| **Beverage substitution effect:** |  |  |  |  |  |
| Wine | 0.95 | (0.83, 1.10) | 0.50 |  |  |
| Beer | 1.03 | (0.90, 1.19) | 0.63 |  |  |
| Spirit | 1.14 | (0.74, 1.76) | 0.56 |  |  |
| **Alcohol consumption by sex** |  |  |  |  |  |
| **Drinking category (females)** |  |  |  |  |  |
| Non-drinker (0g/day) | 1.00 |  |  |  |  |
| Moderate drinker (0-20g/day) | 0.72 | (0.51, 1.03) | 0.07 |  |  |
| Heavy drinker (>20g/day) | 0.58 | (0.35, 0.94) | 0.03 |  |  |
| **Drinking category (males)** |  |  |  |  |  |
| Non-drinker (0g/day) | 1.00 |  |  |  |  |
| Moderate drinker (0-20g/day) | 1.05 | (0.69, 1.59) | 0.83 |  |  |
| Heavy drinker (>20g/day) | 0.74 | (0.47, 1.15) | 0.18 |  |  |
| **Wald test for interaction** |  |  | 0.42 |  |  |
| **Alcohol consumption^#^ (standard drinks per day)** |  |  |  |  |  |
| OR for increase in 1 standard drink per day for women | 0.87 | (0.76, 0.99) | 0.03 |  |  |
| OR for increase of 1 standard drink per day for men | 0.93 | (0.86, 1.01) | 0.09 |  |  |
| **Wald test for interaction** |  |  | 0.33 |  |  |

**ORs adjusted for BMI, age, sex, state, country of birth, and smoking status. ^#^Drinking category defined by average daily ethanol intake in grams. Beverage substitution effect: predicted change in odds associated with substituting one more standard drink per day of this type for one less standard drink of other alcoholic beverages combined. ^#^Alcohol consumption modelled as a continuous variable with 1 standard drink per day (10g ethanol) units*

**Table S2.2. Tobacco smoking and risk of multiple myeloma, adjusted for BMI**

|  | **OR*** | | **(95% CI)** | **P-value** | **Cases** | **Controls** |
| --- | --- | --- | --- | --- | --- | --- |
| **All participants** |  | |  |  | 767 | 1077 |
| **Smoking status** |  | |  |  |  |  |
| *Non-smoker* | 1.00 | |  |  |  |  |
| *Former smoker* | 1.14 | | (0.92, 1.40) | 0.22 |  |  |
| *Current smoker* | 1.25 | | (0.87, 1.81) | 0.23 |  |  |
|  |  | |  |  |  |  |
| **Pack-year history***^#^*  *(mean-centred,*  *per 40 pack-years)* | 1.02 | | (0.76, 1.37) | 0.88 |  |  |
| **Ever-smoker** | 1.16 | | (0.95, 1.41) | 0.14 |  |  |
|  |  | |  |  |  |  |
| **Smoking duration**  *(per 10-year increment)* | | 1.01 | (0.89, 1.14) | 0.86 |  |  |
| **Smoking intensity**  *(per 15 cigarettes/day)* | | 0.99 | (0.81, 1.22) | 0.96 |  |  |
| **Time since cessation**  *0 to <2* | | 1.00 |  |  |  |  |
| *2 to 10* | | 1.25 | (0.78, 1.98) | 0.36 |  |  |
| *10 to 20* | | 1.29 | (0.88, 1.89) | 0.19 |  |  |
| *20 to 30* | | 1.15 | (0.82, 1.60) | 0.43 |  |  |
| *30+ years* | | 1.02 | (0.75, 1.38) | 0.90 |  |  |
| **Age at initiation**  <12 years | | 1.00 |  |  |  |  |
| *12 to 14* | | 0.70 | (0.40, 1.22) | 0.20 |  |  |
| *15 to 19* | | 0.74 | (0.44, 1.25) | 0.26 |  |  |
| *20 to 29* | | 0.86 | (0.52, 1.43) | 0.56 |  |  |
| *30+ years* | | 0.84 | (0.45, 1.57) | 0.59 |  |  |

**Adjusted for BMI, age, sex, state, country of birth, and alcohol consumption. ^#^One pack-year equivalent to smoking 20 cigarettes (i.e., one “pack”) per day for one year, 40 pack-years would be equivalent to doing the same for 40 years (or, e.g., smoking two packs per day for 20 years).*

**Matched and unmatched sensitivity analyses in subsets of EMMA participants**

*The following tables display results of sensitivity analyses for various alcohol and smoking related exposure variables and risk of MM. The analyses are conducted using EMMA participants only:*

*1. Primary analysis – unconditional multivariable regression with all eligible participants,*

*2. Unmatched spouses – unconditional multivariable regression excluding sibling controls,*

*3. Matched siblings – conditional multivariable regression within matched sibships,*

*4. Matched spouses – conditional multivariable regression within matched case-partner pairs:*

| **Smoking Analysis 1 - Smoking status** | | | | |  | |  | |  |  |  |  |  |
| --- | --- | --- | --- | --- | --- | --- | --- | --- | --- | --- | --- | --- | --- |
|  |  |  |  |  | |  | |  |  |  |  |  |  |
| *Primary analysis* | **OR** | **(95% CI)** | **P-value** | **Cases** | | **Controls** | |  |  |  |  |  |  |
| **Smoking status** | |  |  | Cases=791 | | Controls=700 | |  |  |  |  |  |  |
| Non-smoker | 1.00 |  |  |  | |  | |  |  |  |  |  |  |
| Former smoker | 1.15 | (0.92, 1.43) | 0.23 |  | |  | |  |  |  |  |  |  |
| Current smoker | 0.95 | (0.62, 1.47) | 0.82 |  | |  | |  |  |  |  |  |  |
|  |  |  |  |  | |  | |  |  |  |  |  |  |
| *Unmatched spouses* | **OR** | **(95% CI)** | **P-value** | **Cases** | | **Controls** | |  |  |  |  |  |  |
| **Smoking status** | |  |  | Cases=791 | | Controls=285 | |  |  |  |  |  |  |
| Non-smoker | 1.00 |  |  |  | |  | |  |  |  |  |  |  |
| Former smoker | 1.25 | (0.92, 1.70) | 0.16 |  | |  | |  |  |  |  |  |  |
| Current smoker | 1.12 | (0.61, 2.04) | 0.71 |  | |  | |  |  |  |  |  |  |
|  |  |  |  |  | |  | |  |  |  |  |  |  |
| *Matched siblings* | **OR** | **(95% CI)** | **P-value** | **Cases** | | **Controls** | |  |  |  |  |  |  |
| **Smoking status** | |  |  | Cases=311 | | Controls=381 | |  |  |  |  |  |  |
| Non-smoker | 1.00 |  |  |  | |  | |  |  |  |  |  |  |
| Former smoker | 1.19 | (0.83, 1.70) | 0.34 |  | |  | |  |  |  |  |  |  |
| Current smoker | 0.50 | (0.23, 1.08) | 0.08 |  | |  | |  |  |  |  |  |  |
|  |  |  |  |  | |  | |  |  |  |  |  |  |
| *Matched spouses* | **OR** | **(95% CI)** | **P-value** | **Cases** | | **Controls** | |  |  |  |  |  |  |
| **Smoking status** | |  |  | Cases=263 | | Controls=263 | |  |  |  |  |  |  |
| Non-smoker | 1.00 |  |  |  | |  | |  |  |  |  |  |  |
| Former smoker | 1.38 | (0.89, 2.14) | 0.15 |  | |  | |  |  |  |  |  |  |
| Current smoker | 0.80 | (0.30, 2.12) | 0.65 |  | |  | |  |  |  |  |  |  |

| **Smoking Analysis 2 – Mean-centred pack-years (per 40 pack-years) with ever-smoked indicator** | | | | | | |  |  |  |  |
| --- | --- | --- | --- | --- | --- | --- | --- | --- | --- | --- |
| *Primary analysis* | **OR** | **(95% CI)** | **P-value** | **Cases** | **Controls** |  |  |  |  |  |
| **Smoking pack-years (unit: 40 pack-years)** | 0.96 | (0.69, 1.33) | 0.80 | Cases=791 | Controls=700 |  |  |  |  |  |
| **Ever-smoked** | 1.13 | (0.90, 1.41) | 0.30 |  |  |  |  |  |  |  |
|  |  |  |  |  |  |  |  |  |  |  |
| *Unmatched spouses* | **OR** | **(95% CI)** | **P-value** | **Cases** | **Controls** |  |  |  |  |  |
| **Smoking pack-years (unit: 40 pack-years)** | 0.98 | (0.63, 1.53) | 0.92 | Cases=791 | Controls=285 |  |  |  |  |  |
| **Ever-smoked** | 1.23 | (0.90, 1.69) | 0.18 |  |  |  |  |  |  |  |
| *Matched siblings* | **OR** | **(95% CI)** | **P-value** | **Cases** | **Controls** |  |  |  |  |  |
| **Smoking pack-years (unit: 40 pack-years)** | 0.86 | (0.50, 1.48) | 0.59 | Cases=311 | Controls=381 |  |  |  |  |  |
| **Ever-smoked** | 1.12 | (0.78, 1.62) | 0.53 |  |  |  |  |  |  |  |
|  |  |  |  |  |  |  |  |  |  |  |
| *Matched spouses* | **OR** | **(95% CI)** | **P-value** | **Cases** | **Controls** |  |  |  |  |  |
| **Smoking pack-years (unit: 40 pack-years)** | 0.80 | (0.46, 1.41) | 0.45 | Cases=263 | Controls=263 |  |  |  |  |  |
| **Ever-smoked** | 1.36 | (0.88, 2.13) | 0.17 |  |  |  |  |  |  |  |

| **Smoking Analysis 3 - Duration & Intensity** | | | | | |  | |  | |  |  |  |  |  |
| --- | --- | --- | --- | --- | --- | --- | --- | --- | --- | --- | --- | --- | --- | --- |
| *Primary analysis* | **OR** | **(95% CI)** | **P-value** | | **Cases** | | **Controls** | |  |  |  |  |  |  |
| **Smoking duration (units: 10 years)** | 0.87 | (0.68, 1.11) | 0.26 | Cases=354 | | | Controls=297 | |  |  |  |  |  |  |
| **Smoking intensity (unit: 15 cigarettes/day)** | 1.01 | (0.78, 1.30) | 0.96 | Cases=354 | | | Controls=297 | |  |  |  |  |  |  |
|  |  |  |  |  | | |  | |  |  |  |  |  |  |
| *Unmatched spouses* | **OR** | **(95% CI)** | **P-value** | **Cases** | | | **Controls** | |  |  |  |  |  |  |
| **Smoking duration (unit: 10 years)** | 0.64 | (0.43, 0.96) | 0.03 | Cases=354 | | | Controls=110 | |  |  |  |  |  |  |
| **Smoking intensity (unit: 15 cigarettes/day)** | 1.07 | (0.75, 1.53) | 0.71 | Cases=354 | | | Controls=110 | |  |  |  |  |  |  |
|  |  |  |  |  | | |  | |  |  |  |  |  |  |
| *Matched siblings* | **OR** | **(95% CI)** | **P-value** | **Cases** | | | **Controls** | |  |  |  |  |  |  |
| **Smoking duration (unit: 10 years)** | 0.80 | (0.47, 1.36) | 0.41 | Cases=75 | | | Controls=87 | |  |  |  |  |  |  |
| **Smoking intensity (unit: 15 cigs/day)** | 1.59 | (0.89, 2.85) | 0.11 | Cases=75 | | | Controls=87 | |  |  |  |  |  |  |
|  |  |  |  |  | | |  | |  |  |  |  |  |  |
| *Matched spouses* | **OR** | **(95% CI)** | **P-value** | **Cases** | | | **Controls** | |  |  |  |  |  |  |
| **Smoking duration (unit: 10 years)** | 0.73 | (0.39, 1.35) | 0.31 | Cases=60 | | | Controls=60 | |  |  |  |  |  |  |
| **Smoking intensity (unit: 15 cigarettes/day)** | 1.18 | (0.56, 2.52) | 0.66 | Cases=60 | | | Controls=60 | |  |  |  |  |  |  |

| **Smoking Analysis 4 - Age of initiation** | | | | | | |  | | |  | | | | | |  |  |  | |  | |  |  |  |  |
| --- | --- | --- | --- | --- | --- | --- | --- | --- | --- | --- | --- | --- | --- | --- | --- | --- | --- | --- | --- | --- | --- | --- | --- | --- | --- |
| *Primary analysis* | **OR** | | **(95% CI)** | | **P-value** | | **Cases** | | **Controls** | | | |  |  |  |  |  |  |  |  |  |  |  |  |  |
| **Smoking initiation (unit: years)** | 1.04 | | (1.00, 1.08) | | 0.03 | | Cases=354 | | Controls=297 | | | |  |  |  |  |  |  |  |  |  |  |  |  |  |
|  |  | |  | |  | |  | |  | | | |  |  |  |  |  |  |  |  |  |  |  |  |  |
| *Unmatched spouses* | **OR** | | **(95% CI)** | | **P-value** | | **Cases** | | **Controls** | | | |  |  |  |  |  |  |  |  |  |  |  |  |  |
| **Smoking initiation (unit: years)** | 1.08 | | (1.01, 1.15) | | 0.02 | | Cases=354 | | Controls=110 | | | |  |  |  |  |  |  |  |  |  |  |  |  |  |
|  |  | |  | |  | |  | |  | | | |  |  |  |  |  |  |  |  |  |  |  |  |  |
| *Matched siblings* | **OR** | | **(95% CI)** | | **P-value** | | **Cases** | | **Controls** | | | |  |  |  |  |  |  |  |  |  |  |  |  |  |
| **Smoking initiation (unit: years)** | 1.01 | | (0.94, 1.08) | | 0.76 | | Cases=75 | | Controls=87 | | | |  |  |  |  |  |  |  |  |  |  |  |  |  |
|  |  | |  | |  | |  | |  | | | |  |  |  |  |  |  |  |  |  |  |  |  |  |
| *Matched spouses* | **OR** | | **(95% CI)** | | **P-value** | | **Cases** | | **Controls** | | | |  |  |  |  |  |  |  |  |  |  |  |  |  |
| **Smoking initiation (unit: years)** | 1.08 | | (0.94, 1.24) | | 0.30 | | Cases=60 | | Controls=60 | | | |  |  |  |  |  |  |  |  |  |  |  |  |  |
| **Smoking Analysis 5 – Time since cessation** | | | | | | | | | | | |  | | |  | | | |  | |  | |  |  |  |
| *Primary analysis* | | **OR** | | **(95% CI)** | | **P-value** | | **Cases** | | | **Controls** | | |  |  |  |  |  |  |  |  |  |  |  |  |
| **Time since cessation (unit: years)** | | 0.99 | | (0.98, 1.01) | | 0.21 | | Cases=354 | | | Controls=297 | | |  |  |  |  |  |  |  |  |  |  |  |  |
|  | |  | |  | |  | |  | | |  | | |  |  |  |  |  |  |  |  |  |  |  |  |
| **Unmatched spouses** | | **OR** | | **(95% CI)** | | **P-value** | | **Cases** | | | **Controls** | | |  |  |  |  |  |  |  |  |  |  |  |  |
| **Time since cessation (unit: years)** | | 0.98 | | (0.96, 1.00) | | 0.03 | | Cases=354 | | | Controls=110 | | |  |  |  |  |  |  |  |  |  |  |  |  |
|  | |  | |  | |  | |  | | |  | | |  |  |  |  |  |  |  |  |  |  |  |  |
| *Matched siblings* | | **OR** | | **(95% CI)** | | **P-value** | | **Cases** | | | **Controls** | | |  |  |  |  |  |  |  |  |  |  |  |  |
| **Time since cessation (unit: years)** | | 1.01 | | (0.98, 1.04) | | 0.59 | | Cases=75 | | | Controls=87 | | |  |  |  |  |  |  |  |  |  |  |  |  |
|  | |  | |  | |  | |  | | |  | | |  |  |  |  |  |  |  |  |  |  |  |  |
| *Matched spouses* | | **OR** | | **(95% CI)** | | **P-value** | | **Cases** | | | **Controls** | | |  |  |  |  |  |  |  |  |  |  |  |  |
| **Time since cessation (unit: years)** | | 0.99 | | (0.94, 1.04) | | 0.72 | | Cases=60 | | | Controls=60 | | |  |  |  |  |  |  |  |  |  |  |  |  |

| **Smoking Analysis 6 - Intensity & Duration (Restricted to current smokers)** | | | | | | |  |  |  |  |
| --- | --- | --- | --- | --- | --- | --- | --- | --- | --- | --- |
| *Primary analysis* | **OR** | **(95% CI)** | **P-value** | **Cases** | **Controls** |  |  |  |  |  |
| **Smoking intensity (unit: 15 cigarettes/day)** | 0.82 | (0.41, 1.64) | 0.58 | Cases=46 | Controls=49 |  |  |  |  |  |
| **Smoking duration (unit: 10 years)** | 0.80 | (0.51, 1.25) | 0.32 | Cases=46 | Controls=49 |  |  |  |  |  |
|  |  |  |  |  |  |  |  |  |  |  |
| *Unmatched spouses* | **OR** | **(95% CI)** | **P-value** | **Cases** | **Controls** |  |  |  |  |  |
| **Smoking intensity (unit: 15 cigarettes/day)** | 1.00 | (0.35, 2.85) | 0.99 | Cases=46 | Controls=17 |  |  |  |  |  |
| **Smoking duration (unit: 10 years)** | 0.50 | (0.20, 1.23) | 0.13 | Cases=46 | Controls=17 |  |  |  |  |  |

| **Alcohol Analysis 1 – Drinking Category** | | | | | | | | | | | | | | |  |  |  |  |
| --- | --- | --- | --- | --- | --- | --- | --- | --- | --- | --- | --- | --- | --- | --- | --- | --- | --- | --- |
| *Primary analysis* | | | **OR** | **(95% CI)** | | **P-value** | | | **Cases** | | **Controls** |  |  |  |  |  |  |  |
| **Drinking category (Non/Mod./Heavy)** | | |  |  | |  | | Cases=791 | | | Controls=700 |  |  |  |  |  |  |  |
| Non-drinker (0g/day) | | | 1.00 |  | |  | |  | | |  |  |  |  |  |  |  |  |
| Moderate drinker (0-20g/day) | | | 0.81 | (0.60, 1.09) | | 0.16 | |  | | |  |  |  |  |  |  |  |  |
| Heavy drinker (>20g/day) | | | 0.62 | (0.44, 0.87) | | 0.01 | |  | | |  |  |  |  |  |  |  |  |
| *Unmatched spouses* | | | **OR** | **(95% CI)** | | **P-value** | | **Cases** | | | **Controls** |  |  |  |  |  |  |  |
| **Drinking category (Non/Mod./Heavy)** | | |  |  | |  | | Cases=791 | | | Controls=285 |  |  |  |  |  |  |  |
| Non-drinker (0g/day) | | | 1.00 |  | |  | |  | | |  |  |  |  |  |  |  |  |
| Moderate drinker (0-20g/day) | | | 0.84 | (0.56, 1.24) | | 0.38 | |  | | |  |  |  |  |  |  |  |  |
| Heavy drinker (>20g/day) | | | 0.62 | (0.38, 1.00) | | 0.05 | |  | | |  |  |  |  |  |  |  |  |
| *Matched siblings* | | | **OR** | **(95% CI)** | | **P-value** | | **Cases** | | | **Controls** |  |  |  |  |  |  |  |
| **Drinking category (Non/Mod./Heavy)** | | |  |  | |  | | Cases=311 | | | Controls=381 |  |  |  |  |  |  |  |
| Non-drinker (0g/day) | | | 1.00 |  | |  | |  | | |  |  |  |  |  |  |  |  |
| Moderate drinker (0-20g/day) | | | 1.04 | (0.62, 1.75) | | 0.87 | |  | | |  |  |  |  |  |  |  |  |
| Heavy drinker (>20g/day) | | | 0.85 | (0.46, 1.56) | | 0.60 | |  | | |  |  |  |  |  |  |  |  |
| *Matched spouses* | | | **OR** | **(95% CI)** | | **P-value** | | **Cases** | | | **Controls** |  |  |  |  |  |  |  |
| **Drinking category (Non/Mod./Heavy)** | | |  |  | |  | | Cases=263 | | | Controls=263 |  |  |  |  |  |  |  |
| Non-drinker (0g/day) | | | 1.00 |  | |  | |  | | |  |  |  |  |  |  |  |  |
| Moderate drinker (0-20g/day) | | | 0.67 | (0.34, 1.33) | | 0.25 | |  | | |  |  |  |  |  |  |  |  |
| Heavy drinker (>20g/day) | | | 0.54 | (0.23, 1.25) | | 0.15 | |  | | |  |  |  |  |  |  |  |  |
| **Alcohol Analysis 2: Odds ratio for MM and increase of one standard drink per day (units: 10g ethanol/day)** | | | | | | | | | | | | | |  |  |  |  |  |
| *Primary analysis* | **OR** | **(95% CI)** | | | **P-value** | | **Cases** | | | **Controls** | | |  |  |  |  |  |  |
|  | 0.91 | (0.85, 0.98) | | | 0.01 | | Cases=791 | | | Controls=700 | | |  |  |  |  |  |  |
| *Unmatched spouses* | **OR** | **(95% CI)** | | | **P-value** | | **Cases** | | | **Controls** | | |  |  |  |  |  |  |
|  | 0.90 | (0.81, 0.99) | | | 0.03 | | Cases=791 | | | Controls=285 | | |  |  |  |  |  |  |
| *Matched siblings* | **OR** | **(95% CI)** | | | **P-value** | | **Cases** | | | **Controls** | | |  |  |  |  |  |  |
|  | 1.00 | (0.89, 1.13) | | | 0.97 | | Cases=311 | | | Controls=381 | | |  |  |  |  |  |  |
| *Matched spouses* | **OR** | **(95% CI)** | | | **P-value** | | **Cases** | | | **Controls** | | |  |  |  |  |  |  |
|  | 0.84 | (0.70, 1.01) | | | 0.06 | | Cases=263 | | | Controls=263 | | |  |  |  |  |  |  |

| **Alcohol Analysis 3 - Beverage Substitution Analysis*** | | | | | |  | |  |  |  |  |  |
| --- | --- | --- | --- | --- | --- | --- | --- | --- | --- | --- | --- | --- |
|  | | |  |  |  | |  |  |  |  |  |  |
| *Primary analysis* | **OR** | **(95% CI)** | **P-value** | **Cases** | **Controls** | |  |  |  |  |  |  |
| **Wine: substituting 1 std. drink for other alcohol** | 0.93 | (0.80, 1.07) | 0.30 | Cases=791 | Controls=700 | |  |  |  |  |  |  |
| **Beer: substituting 1 std. drink for other alcohol** | 1.07 | (0.92, 1.24) | 0.39 | Cases=791 | Controls=700 | |  |  |  |  |  |  |
| **Spirit: substituting 1 std. drink for other alcohol** | 1.13 | (0.73, 1.76) | 0.58 | Cases=791 | Controls=700 | |  |  |  |  |  |  |
| *Unmatched spouses* | **OR** | **(95% CI)** | **P-value** | **Cases** | **Controls** | |  |  |  |  |  |  |
| **Wine: substituting 1 std. drink for other alcohol** | 1.00 | (0.81, 1.22) | 0.97 | Cases=791 | Controls=285 | |  |  |  |  |  |  |
| **Beer: substituting 1 std. drink for other alcohol** | 0.96 | (0.78, 1.17) | 0.68 | Cases=791 | Controls=285 | |  |  |  |  |  |  |
| **Spirit: substituting 1 std. drink for other alcohol** | 1.68 | (0.78, 3.62) | 0.19 | Cases=791 | Controls=285 | |  |  |  |  |  |  |
| *Matched siblings* | **OR** | **(95% CI)** | **P-value** | **Cases** | **Controls** | |  |  |  |  |  |  |
| **Wine: substituting 1 std. drink for other alcohol** | 0.82 | (0.65, 1.04) | 0.10 | Cases=311 | Controls=381 | |  |  |  |  |  |  |
| **Beer: substituting 1 std. drink for other alcohol** | 1.28 | (1.01, 1.62) | 0.04 | Cases=311 | Controls=381 | |  |  |  |  |  |  |
| **Spirit: substituting 1 std. drink for other alcohol** | 0.44 | (0.17, 1.12) | 0.08 | Cases=311 | Controls=381 | |  |  |  |  |  |  |
| *Matched spouses* | **OR** | **(95% CI)** | **P-value** | **Cases** | **Controls** | |  |  |  |  |  |  |
| **Wine: substituting 1 std. drink for other alcohol** | 0.86 | (0.63, 1.17) | 0.32 | Cases=263 | Controls=263 | |  |  |  |  |  |  |
| **Beer: substituting 1 std. drink for other alcohol** | 1.13 | (0.84, 1.51) | 0.43 | Cases=263 | Controls=263 | |  |  |  |  |  |  |
| **Spirit: substituting 1 std. drink for other alcohol** | 1.43 | (0.47, 4.35) | 0.53 | Cases=263 | Controls=263 | |  |  |  |  |  |  |

*Results stem from 3 separate models by beverage and the Wald test for coefficients, e.g. (Beer) - (Not beer) = 0

| **Alcohol Analysis 4: Interaction drinking category & sex** | | | | | | | | |  |  | | | | |  |  |
| --- | --- | --- | --- | --- | --- | --- | --- | --- | --- | --- | --- | --- | --- | --- | --- | --- |
| *Primary analysis* | | | | **OR** | **(95% CI)** | | **P-value** | **Cases** | | | **Controls** | | |  |  |  |
| **Drinking category (males)** | | | |  |  | |  | Cases=791 | | | Controls=700 | | |  |  |  |
| Non | | | | 1.00 |  | |  |  | | |  | | |  |  |  |
| Moderate | | | | 0.95 | (0.60, 1.52) | | 0.83 |  | | |  | | |  |  |  |
| Heavy | | | | 0.74 | (0.46, 1.21) | | 0.23 |  | | |  | | |  |  |  |
| **Drinking category (females)** | | | |  |  | |  |  | | |  | | |  |  |  |
| Non | | | | 1.00 |  | |  |  | | |  | | |  |  |  |
| Moderate | | | | 0.73 | (0.50, 1.06) | | 0.10 |  | | |  | | |  |  |  |
| Heavy | | | | 0.52 | (0.31, 0.88) | | 0.01 |  | | |  | | |  |  |  |
| **Wald test for interaction (sex#drinking)** | | | |  |  | | 0.61 |  | | |  | | |  |  |  |
| *Unmatched spouses* | | | | **OR** | **(95% CI)** | | **P-value** | **Cases** | | | **Controls** | | |  |  |  |
| **Drinking category (males)** | | | |  |  | |  | Cases=791 | | | Controls=285 | | |  |  |  |
| Non | | | | 1.00 |  | |  |  | | |  | | |  |  |  |
| Moderate | | | | 1.03 | (0.52, 2.06) | | 0.93 |  | | |  | | |  |  |  |
| Heavy | | | | 0.68 | (0.33, 1.42) | | 0.31 |  | | |  | | |  |  |  |
| **Drinking category (females)** | | | |  |  | |  |  | | |  | | |  |  |  |
| Non | | | | 1.00 |  | |  |  | | |  | | |  |  |  |
| Moderate | | | | 0.76 | (0.47, 1.22) | | 0.26 |  | | |  | | |  |  |  |
| Heavy | | | | 0.63 | (0.33, 1.20) | | 0.16 |  | | |  | | |  |  |  |
| **Wald test for interaction (sex#drinking)** | | | |  |  | | 0.71 |  | | |  | | |  |  |  |
| *Matched siblings* | | | | **OR** | **(95% CI)** | | **P-value** | **Cases** | | | **Controls** | | |  |  |  |
| **Drinking category (males)** | | | |  |  | |  | Cases=311 | | | Controls=381 | | |  |  |  |
| Non | | | | 1.00 |  | |  |  | | |  | | |  |  |  |
| Moderate | | | | 1.02 | (0.49, 2.10) | | 0.96 |  | | |  | | |  |  |  |
| Heavy | | | | 0.91 | (0.41, 2.03) | | 0.82 |  | | |  | | |  |  |  |
| **Drinking category (females)** | | | |  |  | |  |  | | |  | | |  |  |  |
| Non | | | | 1.00 |  | |  |  | | |  | | |  |  |  |
| Moderate | | | | 1.08 | (0.53, 2.18) | | 0.84 |  | | |  | | |  |  |  |
| Heavy | | | | 0.75 | (0.31, 1.79) | | 0.52 |  | | |  | | |  |  |  |
| **Wald test for interaction (sex#drinking)** | | | |  |  | | 0.81 |  | | |  | | |  |  |  |
| *Matched spouses* | | | | **OR** | **(95% CI)** | | **P-value** | **Cases** | | | **Controls** | | |  |  |  |
| **Drinking category (males)** | | | |  |  | |  | Cases=263 | | | Controls=263 | | |  |  |  |
| Non | | | | 1.00 |  | |  |  | | |  | | |  |  |  |
| Moderate | | | | 0.70 | (0.27, 1.78) | | 0.45 |  | | |  | | |  |  |  |
| Heavy | | | | 0.54 | (0.20, 1.48) | | 0.23 |  | | |  | | |  |  |  |
| **Drinking category (females)** | | | |  |  | |  |  | | |  | | |  |  |  |
| Non | | | | 1.00 |  | |  |  | | |  | | |  |  |  |
| Moderate | | | | 0.65 | (0.31, 1.36) | | 0.25 |  | | |  | | |  |  |  |
| Heavy | | | | 0.56 | (0.20, 1.58) | | 0.27 |  | | |  | | |  |  |  |
| **Wald test for interaction (sex#drinking)** | | | |  |  | | 0.97 |  | | |  | | |  |  |  |
| NB: Non-drinking 0g ethanol/day, Moderate-drinking 0-20g/d, Heavy-drinking 20g+/d | | | | | | | | | | | | | |  |  |  |
| **Alcohol Analysis 5 - Interaction sex # ethanol intake (continuous)** | | | | | | | | | | | | |  |  |  |  |
| *Primary analysis* | **OR** | **(95% CI)** | **P-value** | | **Cases** | **Controls** | | | | | |  |  |  |  |  |
| **Men** | 0.96 | (0.88, 1.04) | 0.33 | | Cases=791 | Controls=700 | | | | | |  |  |  |  |  |
| **Women** | 0.83 | (0.72, 0.95) | 0.01 | | Cases=791 | Controls=700 | | | | | |  |  |  |  |  |
| **Wald test for interaction** |  |  | 0.08 | |  |  | | | | | |  |  |  |  |  |
| *Unmatched spouses* | **OR** | **(95% CI)** | **P-value** | | **Cases** | **Controls** | | | | | |  |  |  |  |  |
| **Men** | 0.93 | (0.82, 1.06) | 0.26 | | Cases=791 | Controls=285 | | | | | |  |  |  |  |  |
| **Women** | 0.85 | (0.73, 1.00) | 0.05 | | Cases=791 | Controls=285 | | | | | |  |  |  |  |  |
| **Wald test for interaction** |  |  | 0.40 | |  |  | | | | | |  |  |  |  |  |
| *Matched siblings* | **OR** | **(95% CI)** | **P-value** | | **Cases** | **Controls** | | | | | |  |  |  |  |  |
| **Men** | 1.07 | (0.93, 1.23) | 0.36 | | Cases=311 | Controls=381 | | | | | |  |  |  |  |  |
| **Women** | 0.87 | (0.72, 1.07) | 0.19 | | Cases=311 | Controls=381 | | | | | |  |  |  |  |  |
| **Wald test for interaction** |  |  | 0.09 | |  |  | | | | | |  |  |  |  |  |
| *Matched spouses* | **OR** | **(95% CI)** | **P-value** | | **Cases** | **Controls** | | | | | |  |  |  |  |  |
| **Men** | 0.85 | (0.71, 1.03) | 0.10 | | Cases=263 | Controls=263 | | | | | |  |  |  |  |  |
| **Women** | 0.71 | (0.52, 0.97) | 0.03 | | Cases=263 | Controls=263 | | | | | |  |  |  |  |  |
| **Wald test for interaction** |  |  | 0.18 | |  |  | | | | | |  |  |  |  |  |

**OR for increase of 1 standard drink per day*

## Supporting information 3: Directed Acyclic Graphs (DAG)

**DAG1: Smoking and multiple myeloma**


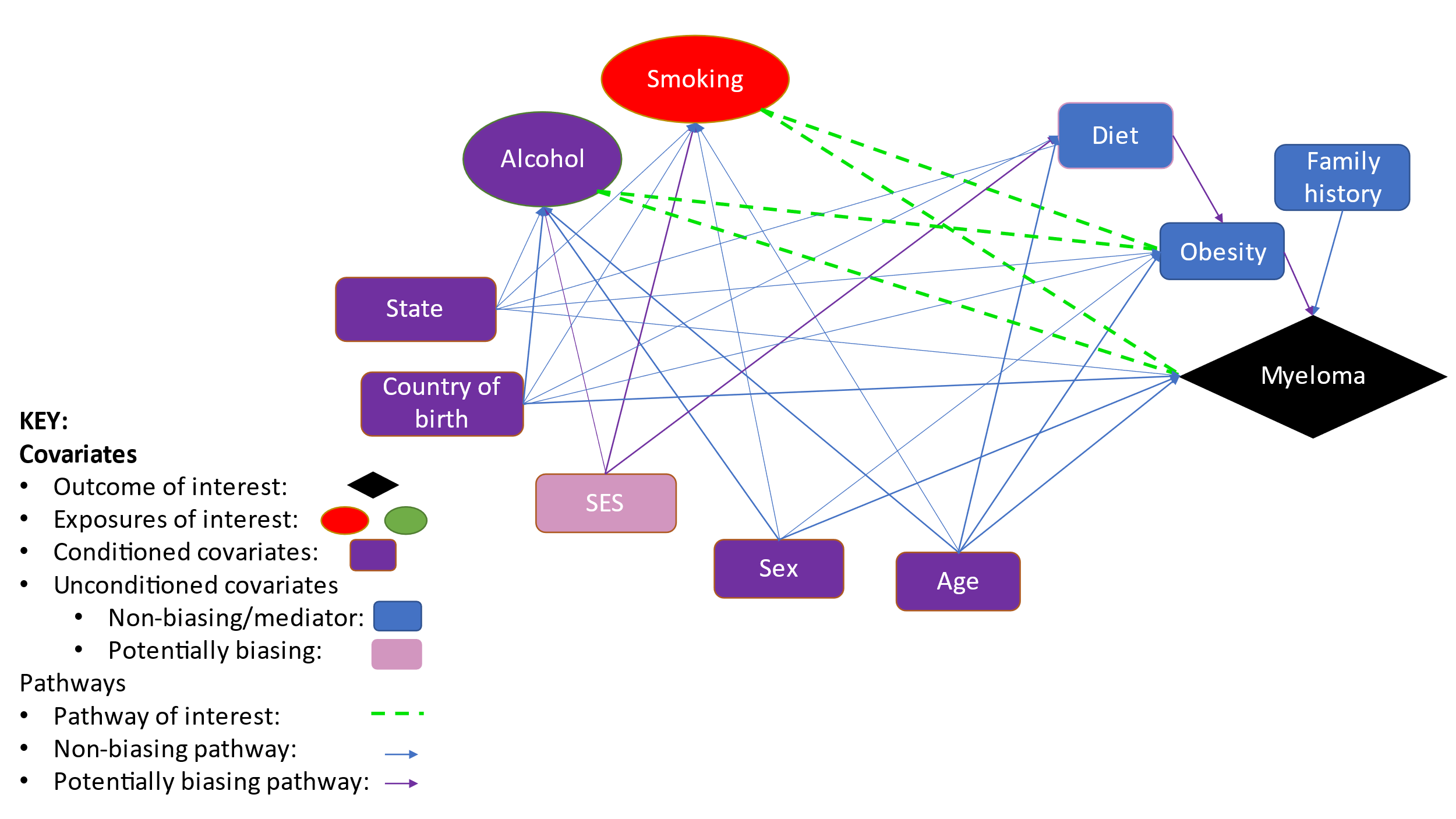


**DAG2: Alcohol and multiple myeloma**


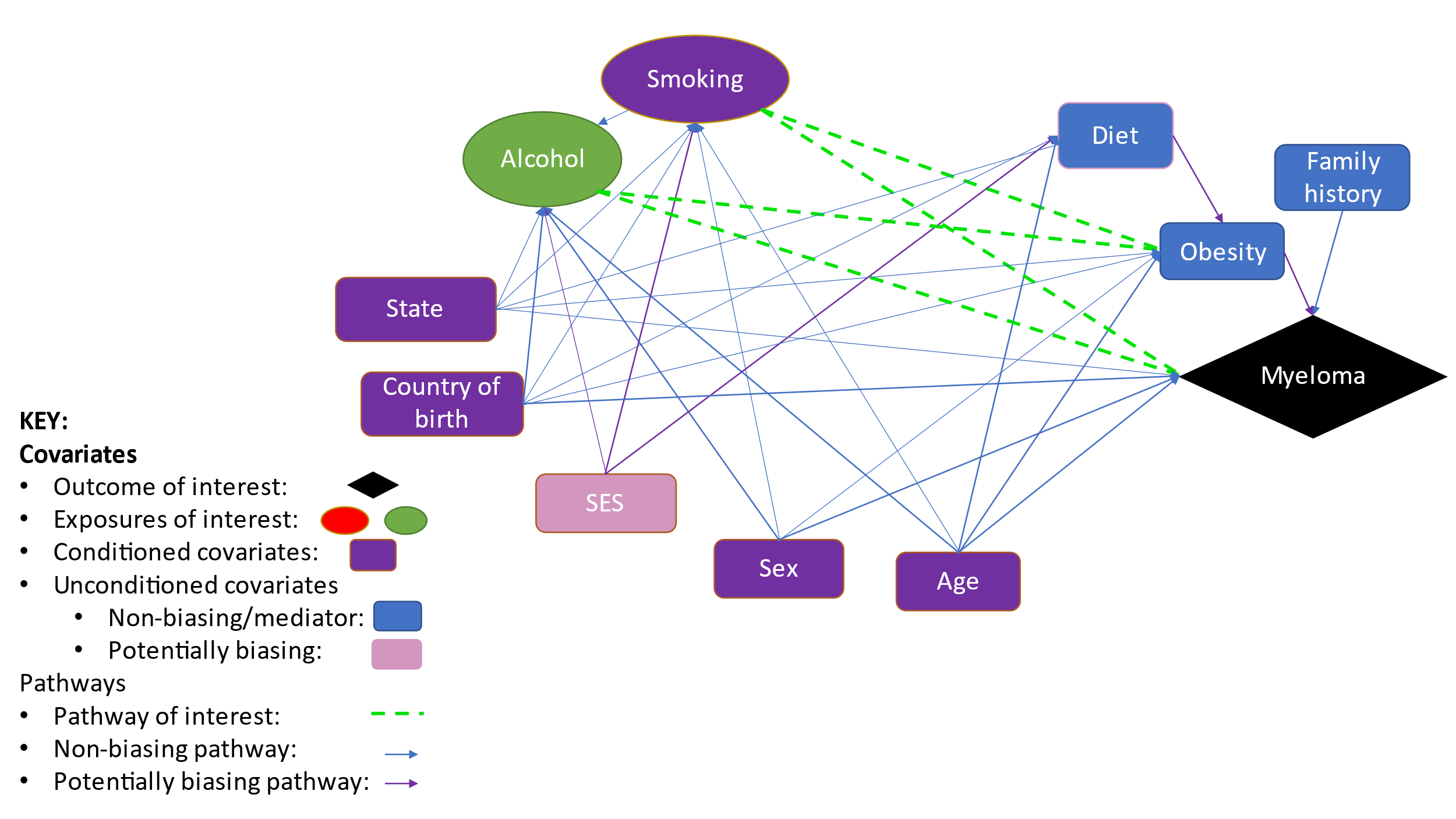


Papers supporting inclusion of variables in the above DAGs:

1. Alexander DD, Mink PJ, Adami H, Cole P, Mandel JS, Oken MM, et al. Multiple myeloma: a review of the epidemiologic literature. International Journal of Cancer. 2007;120(S12):40–61.
2. Cowan AJ, Allen C, Barac A, Basaleem H, Bensenor I, Curado MP, et al. Global burden of multiple myeloma: a systematic analysis for the global burden of disease study 2016. JAMA Oncology. 2018 Sep 1;4(9):1221–7.
3. Australian Institute of Health and Welfare. Cancer in Australia 2019. Canberra: AIHW; 2019.
4. Traversy G, Chaput J-P. Alcohol consumption and obesity: An update. Current Obesity Reports. 2015 März;4(1):122–30.
5. Sayon-Orea C, Martinez-Gonzalez MA, Bes-Rastrollo M. Alcohol consumption and body weight: a systematic review: Nutrition Reviews, Vol. 69, No. 8. Nutrition Reviews. 2011 Aug;69(8):419–31.
6. Tian J, Venn A, Otahal P, Gall S. The association between quitting smoking and weight gain: a systemic review and meta-analysis of prospective cohort studies. Obesity Reviews. 2016 Oct;17(10):1014–1014.
7. Kino, S., Bernabé, E. & Sabbah, W. Socioeconomic inequality in clusters of health-related behaviours in Europe: latent class analysis of a cross-sectional European survey. BMC Public Health 17, 497 (2017).
8. Méjean C, Droomers M, Van Der Schouw YT, Sluijs I, Czernichow S, Grobbee DE, Bueno-de-Mesquita HB, Beulens JW. The contribution of diet and lifestyle to socioeconomic inequalities in cardiovascular morbidity and mortality. International Journal of Cardiology. 2013 Oct 15;168(6):5190-5.
9. Australian Institute of Health and Welfare 2021. Alcohol, tobacco & other drugs in Australia. Cat. no. PHE 221. Canberra: AIHW. Viewed 24 May 2021,
10. Barsties LS, Walsh SD, Huijts T, Bendtsen P, Molcho M, Buijs T, Vieno A, Elgar FJ, Stevens GW. Alcohol consumption among first‐and second‐generation immigrant and native adolescents in 23 countries: Testing the importance of origin and receiving country alcohol prevalence rates. Drug and Alcohol Review. 2017 Nov;36(6):769-78.
11. Stanaway, FF, Ribeiro, RV, Khalatbari-Soltani, S. et al. Diet quality in an ethnically diverse population of older men in Australia. European Journal of Clinical Nutrition. (2021).
12. Haregu TN, Nanayakkara S, Carrington M, Kaye D. Prevalence and correlates of normal body mass index central obesity among people with cardiovascular diseases in Australia. Public Health. 2020 Jun 1;183:126-31.
13. Hardy, LL, Jin, K, Mihrshahi, S. et al. Trends in overweight, obesity, and waist-to-height ratio among Australian children from linguistically diverse backgrounds, 1997 to 2015. International Journal of Obesity. 43, 116–124 (2019).
14. El Masri A, Kolt GS, George ES. Country of birth differences in lifestyle‐related chronic disease among middle‐aged and older adults of Lebanese ethnicity. Australian and New Zealand Journal of Public Health. 2019 Oct;43(5):429-35.
15. Chaiyasong S, Huckle T, Mackintosh AM, Meier P, Parry CD, Callinan S, Viet Cuong P, Kazantseva E, Gray‐Phillip G, Parker K, Casswell S. Drinking patterns vary by gender, age and country‐level income: Cross‐country analysis of the International Alcohol Control Study. Drug and Alcohol Review. 2018 Aug;37:S53-62.
16. Wensink M, Alvarez JA, Rizzi S, Janssen F, Lindahl-Jacobsen R. Progression of the smoking epidemic in high-income regions and its effects on male-female survival differences: a cohort-by-age analysis of 17 countries. BMC Public Health. 2020 Dec;20(1):1-8.
17. Reitsma MB, Fullman N, Ng M , et al. Smoking prevalence and attributable disease burden in 195 countries and territories, 1990-2015: a systematic analysis from the global burden of disease study 2015. Lancet. 2017;389:1885–906.
18. Melikian AA, Djordjevic MV, Hosey J, et al. Gender differences relative to smoking behavior and emissions of toxins from mainstream cigarette smoke. Nicotine and Tobacco Research. 2007;9:377–87.
19. Hiza HA, Casavale KO, Guenther PM, Davis CA. Diet quality of Americans differs by age, sex, race/ethnicity, income, and education level. Journal of the Academy of Nutrition and Dietetics. 2013 Feb 1;113(2):297-306.

## Supporting information 4: Covariate-MM associations

**Table S4.1. Age, sex, country of birth and odds of multiple myeloma**

|  | **Adjusted OR*** | **(95% CI)** | **Cases (%)** | **Controls (%)** |
| --- | --- | --- | --- | --- |
| **Total** |  |  | 789 (41) | 1113 (59) |
| **Age (per 10 years)** | 1.16 | (1.05, 1.28) |  |  |
| **Sex:** |  |  |  |  |
| Female | 1.00 | Reference |  |  |
| Male | 1.97 | (1.63, 2.39) |  |  |
| **COB:** |  |  |  |  |
| Australia/New Zealand | 1.00 | Reference |  |  |
| Europe or UK | 1.61 | (1.26, 2.07) |  |  |
| Other | 2.20 | (1.50, 3.22) |  |  |

**Adjusted for alcohol consumption (drinking category), smoking status (never/former/current), state and age.*
